# Supplementary material for: A studyforrest extension, MEG recordings while watching the audio-visual movie “Forrest Gump”
Source: Sci Data. 2022 May 13;9:206. doi: 10.1038/s41597-022-01299-1 (PMC9106652; doi:10.1038/s41597-022-01299-1)
Supplement: Supplementary file 2 — Head motion magnitude for each participant [file 41597_2022_1299_MOESM2_ESM.docx]

### Supplementary Information

Head motion magnitude for each participant 2

**
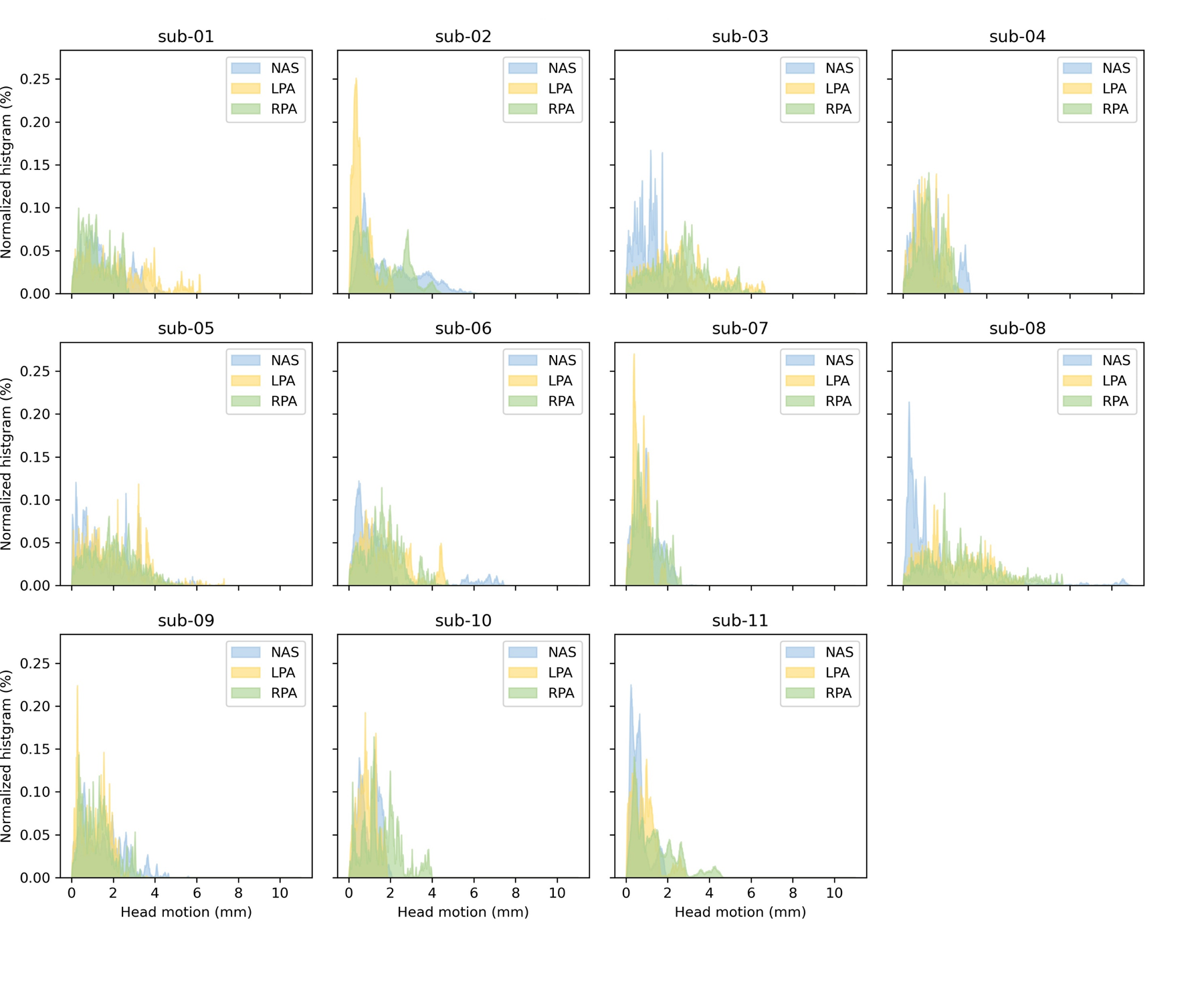
**

**Supplementary Figure 1.** Head motion magnitude from each individual participant. The density histogram of motion magnitude calculated for three fiducials (NAS, LPA, RPA) were plotted for all samples, across all runs for each participant.
